# Chronic Pain: How Challenging Are DDIs in the Analgesic Treatment of Inpatients with Multiple Chronic Conditions?

**DOI:** 10.1371/journal.pone.0168987

**Published:** 2017-01-03

**Authors:** Klarissa Siebenhuener, Emmanuel Eschmann, Alexander Kienast, Dominik Schneider, Christoph E. Minder, Reinhard Saller, Lukas Zimmerli, Jürg Blaser, Edouard Battegay, Barbara M. Holzer

**Affiliations:** 1 Department of Internal Medicine, University Hospital Zurich, Zurich, Switzerland; 2 Center of Competence Multimorbidity, University of Zurich, Zurich, Switzerland; 3 Research Center for Medical Informatics, Directorate of Research and Education, University Hospital Zurich, Zurich, Switzerland; 4 Maennedorf Hospital, Department of Internal Medicine, Canton Zurich, Switzerland; 5 Horten Center, University Hospital Zurich, Zurich, Switzerland; 6 Institute of Complementary and Integrative Medicine, University Hospital Zurich, Zurich, Switzerland; 7 Cantonal Hospital, Internal Medicine, Olten, Switzerland; 8 University Research Priority Program ‘Dynamics of Healthy Aging,’ University of Zurich, Zurich, Switzerland; University of Würzburg, GERMANY

## Abstract

**Background:**

Chronic pain is common in multimorbid patients. However, little is known about the implications of chronic pain and analgesic treatment on multimorbid patients. This study aimed to assess chronic pain therapy with regard to the interaction potential in a sample of inpatients with multiple chronic conditions.

**Methods and Findings:**

We conducted a retrospective study with all multimorbid inpatients aged ≥18 years admitted to the Department of Internal Medicine of University Hospital Zurich in 2011 (n = 1,039 patients). Data were extracted from the electronic health records and reviewed. We identified 433 hospitalizations of patients with chronic pain and analyzed their combinations of chronic conditions (multimorbidity). We then classified all analgesic prescriptions according to the World Health Organization (WHO) analgesic ladder. Furthermore, we used a Swiss drug-drug interactions knowledge base to identify potential interactions between opioids and other drug classes, in particular coanalgesics and other concomitant drugs. Chronic pain was present in 38% of patients with multimorbidity. On average, patients with chronic pain were aged 65.7 years and had a mean number of 6.6 diagnoses. Hypertension was the most common chronic condition. Chronic back pain was the most common painful condition. Almost 90% of patients were exposed to polypharmacotherapy. Of the chronic pain patients, 71.1% received opioids for moderate to severe pain, 43.4% received coanalgesics. We identified 3,186 potential drug-drug interactions, with 17% classified between analgesics (without coanalgesics).

**Conclusions:**

Analgesic drugs-related DDIs, in particular opioids, in multimorbid patients are often complex and difficult to assess by using DDI knowledge bases alone. Drug-multimorbidity interactions are not sufficiently investigated and understood. Today, the scientific literature is scarce for chronic pain in combination with multiple coexisting medical conditions and medication regimens. Our work may provide useful information to enable further investigations in multimorbidity research within the scope of potential interactions and chronic pain.

## Introduction

Pain is a medical condition listed among the most common diseases worldwide. The most common causes of chronic pain relate to musculoskeletal disorders [1]. Prevalence estimates for musculoskeletal pain in elderly patients vary widely, from 32.9% to 60% in Europe [2, 3]. Typically, many people with musculoskeletal pain are multimorbid and receive polypharmacotherapy [4, 5]. Previous, studies on multimorbidity have reported chronic pain diagnoses and treatments as an outcome of only secondary interest—for instance, as a medical condition co-occurring with somatic and mental health disorders [6–9]. Opioids are a mainstay of chronic pain treatment according to the World Health Organization (WHO) three-step analgesic ladder, which was developed in the mid-1980s as a scheme for patients with cancer pain [10]. Meanwhile, opioids have become increasingly popular as a treatment option for patients with chronic non-cancer pain [11, 12], and the range of applications of the WHO analgesic ladder has been extended accordingly.

The WHO scheme also introduced the term “adjuvant drugs”; originally, a small number of drugs (e.g., anxiolytics) was described as adjuvants, to enhance the analgesic “three-step” sequence from non-opioids, to weak opioids, and finally to strong opioids [13]. Later, the scope of these drugs was extended [14]. Key recommendations for adjuvant drugs, as described in the WHO analgesic ladder approach, were to treat adverse effects of analgesics (e.g., antiemetics or laxatives), to enhance pain relief (e.g., corticosteroids in spinal nerve compression), and to treat concomitant psychological comorbidities (e.g., psychotropic drugs).

Today, adjuvant or coanalgesic drugs have become established in the treatment of cancer pain and non-cancer pain. As a consequence, the broad range of alternative analgesic strategies increases the risk of polypharmacotherapy. From this perspective, chronic pain therapy in patients with multiple chronic conditions can be challenging [15]. Little is known, on the one hand, about the clinical impact of opioids and potential adverse drug-drug interactions (DDIs) in multimorbid patients [16], and, on the other hand, about the clinical impact of drug-disease interactions in combination with coanalgesics in patients with chronic pain and multimorbidity.

The primary aim of this study was to assess drugs used to treat pain in multimorbid patients at a tertiary teaching hospital. The secondary aim was to examine the potential of DDIs by using an electronic DDI knowledge base.

We undertook the following steps:

1. We explored the current literature on common chronic conditions and their potential to modify chronic pain.
2. We identified and characterized the target population from patients’ electronic medical chart reviews;
3. We examined and described analgesic prescriptions corresponding to the WHO analgesic pain ladder;
4. We examined and described coanalgesic and concomitant drugs of the target population;
5. We reviewed the potential for DDIs; and

## Material and Methods

### Data Collection

The University Hospital Zurich is a tertiary teaching hospital with 850 beds and about 38,000 inpatient admissions per year. We used a retrospective data set of all multimorbid inpatients admitted to the Department of Internal Medicine in 2011 (n = 1,139 hospitalizations). We included all adult inpatients discharged between January 1 and December 31, 2011, aged 18 years and older, and with more than one chronic medical condition. Patients in a methadone program, pregnant women, and palliative care patients were excluded. This study was conducted in accordance with the Strengthening the Reporting of Observational Studies in Epidemiology (STROBE) guidelines [17].

Data were extracted from the hospital’s electronic health records by the hospital’s Research Center for Medical Informatics. The following variables were extracted and transferred to a spreadsheet (Microsoft Excel® 2010, www.microsoft.com): date of admission, gender, age (years), length of hospital stay (days), all chronic pain diagnoses, all diagnoses of other chronic conditions, and all medications prescribed during hospitalization. Two physicians reviewed each case with regard to the inclusion and exclusion criteria and completed a list of diagnoses, using the patient’s care and discharge report. Patients were categorized as ‘with’ or ‘without’ a painful chronic condition. The diagnoses identified were marked as active if the painful condition required the prescription of analgesics during the patient’s hospital stay. In addition, all analgesic prescriptions were assessed in accordance with the WHO analgesic ladder approach [14]. All chronic conditions were transformed from the ICD-10 to ICPC-2 standard based on the list of chronic conditions provided by O’Halloran et al. [18]. Some ICPC-2 codes were grouped together—for example, the diagnosis of cancer (including solid malignant tumors of different types). All relevant patient charts were individually reviewed and completed by members of the research team. Unclear information about a patient record was reviewed by the study group and agreed on before coding. In a first step we looked at our patient population with regard to multimorbidity and the most frequent combinations of single diseases (triplets), including the drugs prescribed during their hospital stay. In a second step, we classified all prescribed analgesics according to the single steps of the WHO analgesic ladder.

#### Drug-drug interaction knowledge base

Potential interactions between opioids, coanalgesics and other drug classes were identified using the Swiss drug-drug interaction knowledge base galdat/hospINDEX® (distributed by e-mediat AG, Berne, Switzerland; derived from ABDATA Pharma-Daten, Eschborn, Germany), The knowledge base is used at the University Hospital Zurich (at present hospINDEX 2016–05)[19]. This knowledge base categorizes DDIs by severity from level 1 (contraindicated) to level 6 (no action required), according to the ORCA criteria (operational classification of drug interactions, with a focus on clinically relevant interacting drug pairs) [20]. The treatment recommendations presented are collected from clinical practice guidelines, pharmaceutical drug information, or case reports, and they include country-specific information. The frequency of potential DDIs was calculated as the total number of overlapping drug orders identified as interacting according to the knowledge base. In detail, potential DDIs were described separately for opioid-related DDIs and for coanalgesic or concomitant drugs.

### Definitions

#### Multimorbidity

Multimorbidity was defined as the co-occurrence of two or more chronic medical conditions [21, 22]. Chronic conditions were defined following O’Halloran et al. [18], based on the International Classification of Primary Care, second edition (ICPC-2) disease classification system.

#### Chronic pain

The International Association for the Study of Pain defined chronic pain as “pain that persists beyond the normal time of healing” [23]. We defined the persistence of pain for more than three months as chronic. We defined DDIs as a modification of the effect of a drug or as an adverse effect resulting from concomitant administration of one or more other drugs. We refer to potential DDIs only, as actual adverse drug events were not considered in this study. Drug-disease interactions were defined as a modification in the endogenous regulatory system, either in an already pathologically altered or in a hitherto unaffected regulatory system.

#### Adjuvants/coanalgesics

The terms ‘adjuvant drugs,’ ‘adjuvants,’ ‘adjuvant analgesics’ [24–26], ‘coanalgesics,’ or ‘concomitant medication’ in persistent opioid use [27, 28] have been used simultaneously and interchangeably over the past two decades. To date, there is still no consensus in the literature on how to classify the growing number of such agents [29]. Criteria for a meaningful classification have been recommended by Lussier et al. [30]; however, these are applied mainly in the context of cancer pain: adjuvant analgesics were defined as drugs with a primary indication other than pain but having analgesic properties in some painful conditions. From this perspective, adjuvants are potentially beneficial as analgesics, and as a result, the categorization is based on how they are used in clinical practice. Coanalgesics have since become established for the treatment of cancer pain as well as non-cancer pain.

### Chronic Pain Treatment and the WHO Analgesic Ladder

Patients’ pain medications were specified by trade name, using the Anatomical Therapeutic Chemical Classification System code; the duration (in days) of the prescription was also determined. Analgesic prescriptions were assigned to the steps of the WHO analgesic ladder [14]: non-opioid analgesics (step I), weak opioids (step II), and strong opioids (step III). This classification of analgesics is conventionally based on their activity at opioid receptors as either non-opioid or opioid. To characterize all relevant groups with an analgesic effect we used three terms to distinguish between these groups: (1) analgesics–drugs for pain relief based on the WHO analgesic ladder (non-opioids, weak and strong opioids) ‒ i.e., paracetamol (acetaminophen) as a non-opioid; (2) coanalgesics–drugs that are distinctly prescribed in the specific context of pain relief; i.e., pregabalin approved for the treatment of neuropathic pain; and (3) concomitant medication–concurrent use of any drugs that are given in the context of multimorbidity. In some cases nevertheless they may have a modifying effect on pain ‒ i.e., antidepressants for coexisting depression.

### Data Management and Statistical Analysis

For the statistical analyses, we used Stata® statistical software (Version 13, Stata Corporation, College Station, TX; www.stata.com). Continuous data were presented as means with standard deviations, categorical data as counts and proportions. We used t-tests to compare continuous data and chi square tests to compare categorical data. The significance level was set at 0.05.

### Ethics Approval

Ethics approval for this study was obtained from the Research Ethics Committee of the Canton of Zurich, KEK-ZH-No. 2012–0237, which waived the requirement for obtaining informed consent for this retrospective review of electronic medical records. All patient records and information were anonymized and de-identified before the analysis.

## Results

### Study Population and Diagnoses

The prevalence of chronic pain found among the 1039 multimorbid inpatients was 38% (n = 393). On average, these patients were 66 years old, with no significant difference in age between multimorbid patients with and without chronic pain (p = 0.318; Table 1). Multimorbid patients with chronic pain (CP) had significantly more chronic diagnoses (with a mean of 6.6 chronic diagnoses, with 4.4 different organ systems involved) compared with multimorbid patients without chronic pain (mean of 5.0 conditions and 3.1 organ systems involved) (p < 0.00005). Moreover, patients with CP had significantly longer hospital stays than multimorbid patients without CP (15.5 vs. 12.5 days, p = 0.0007).

**Table 1:** Sociodemographic characteristics of the study population (n = 1039): Comparison of multimorbid patients with and without chronic pain in a population of inpatients in a department of internal medicine in a tertiary care hospital.

**Patients**	**Multimorbid patients with chronic pain (n = 393)**	**Multimorbid patients without chronic pain (n = 646)**	**p-value**
**Gender, n (%)**			p = 0.029
Male	202 (51.4)	377 (58.4)	
Female	191 (48.6)	269 (41.6)	
**Mean age, in years (SD**^a^**)**	65.7 (15.9)	66.7 (15.5)	p = 0.318
**Hospitalization**			p = 0.710
Single hospitalization in 2011 (%)	358 (91.1)	584 (90.4)	
Multiple hospitalization in 2011 (%)	35 (8.9)	62 (9.6)	
**Cases**	**Multimorbid patients with chronic pain (n = 433)**	**Multimorbid patients without chronic pain (n = 706)**	**p-value**
**Mean number of chronic conditions (SD)**	6.6 (2.9)	5.01 (2.4)	p < 0.00005
**Mean days of stay in hospital (SD)**	15.7 (19.1)	12.5 (11.6)	p < 0.0007

^a^ SD = Standard deviation.

Hypertension, chronic kidney disease, and diabetes were the most frequent single chronic conditions in both groups, with prevalence estimates for the group with CP of 58.7% for hypertension, 29.1% for chronic kidney disease, and 23.1% for diabetes (data not shown). When only patients with chronic pain diagnoses were considered (Table 2), the most common triple combination was hypertension, chronic back pain, and chronic kidney disease (Table 3). Further, 15.3% of the patients with CP had depressive disorders, which is almost twice as many as the multimorbid patients without CP who had depressive disorders (p < 0.00005). Logistic regression showed that this difference in the prevalence of depression was not explained by either different numbers of chronic conditions or differences in the duration of hospitalization. After adjustment, the difference in the probability of depression between pain and non-pain patients remained significant (p = 0.008).

**Table 2:** Top 10 chronic pain conditions/chronic conditions associated with chronic pain respectively in a population of inpatients at a department of internal medicine in a tertiary care hospital (n = 433 hospitalizations). Multiple counts were allowed.

Most frequent chronic pain diagnoses	Prevalence in %
Chronic back pain	38.8
Osteoarthrosis (joint disorders)	17.6
Cancer	16.6
Rheumatoid/seropositive arthritis	8.1
Musculoskeletal diseases, other^a^	6.9
Cholecystitis/cholelithiasis	6
Osteoporosis	5.3
Chronic enteritis/ulcerative colitis	4.8
Gout	4.2
Neurological diseases	3.9

^a^ This diagnosis includes specific arthropathies, e.g., crystal- related arthropathies.

**Table 3:** The most common triplets of chronic conditions including chronic pain diagnoses in a population of inpatients at a tertiary department of internal medicine (n = 433 hospitalizations).

Chronic conditions			Prevalence in %
Chronic back pain	Hypertension	Chronic kidney disease	9.9
Chronic back pain	Hypertension	Ischemic heart diseases	7.9
Chronic back pain	Hypertension	Diabetes	7.2
Osteoarthrosis	Hypertension	Chronic kidney disease	7.2
Chronic back pain	Hypertension	Osteoarthrosis	6.5
Chronic back pain	Hypertension	Peripheral neuritis/neuropathy	6.0
Chronic back pain	Hypertension	Atrial fibrillation/flutter	5.5
Chronic back pain	Hypertension	Arteriosclerosis	5.3
Osteoarthrosis	Hypertension	Diabetes	5.1
Chronic back pain	Ischemic heart diseases	Chronic kidney disease	5.1

Of all patients with CP, 60% had one, 27% had two, and 13% had three or more pain diagnoses, respectively. The most common chronic pain diagnosis was chronic back pain (38.6%; Table 2), followed by chronic pain diagnoses, such as osteoarthrosis (17.6%), and painful conditions subsequent to cancer (16.6%). Of the 10 most frequent pain diagnoses, seven were related to chronic musculoskeletal diseases.

### Pain Treatment and WHO Analgesic Ladder

The most frequent mode of prescription in terms of assignment to the WHO analgesic ladder was a combination of step I analgesics (non-opioids) with step III analgesics (strong opioids) in 35% of all CP hospitalizations with no step II analgesics (weak opioids). The second most frequent mode was a prescription of step I analgesics only (29%), followed by (third) concurrent prescription of all three steps (19%). Step I analgesics were prescribed to 98.6% of all inpatients with CP, with paracetamol (acetaminophen) or metamizole (novaminsulfone) the most often prescribed (Table 4). In total, 71.1% of these CP patients received opioids, in different combinations, for moderate to severe pain. The most frequent combination with a longer (more than a day) simultaneous prescription of weak and strong opioids was tramadol and morphine. All of these 58 prescriptions were additionally complemented with step I analgesics. In 21 of these 58 simultaneous opioid medications, an upward change took place (stronger medications were required over time), and consequently only strong opioids were prescribed.

**Table 4:** Drug prescriptions in multimorbid patients with chronic pain corresponding to the classification by the WHO analgesic ladder.

WHO analgesic ladder	No. of cases (n = 433)	No. of cases in %	Most frequently prescribed analgesics
**Step I only**	125	28.9	paracetamol, metamizole, NSAIDs^a^
**Step II only**	2	0.5	tramadol*
**Step III only**	4	0.9	morphine, oxycodone, fentanyl transdermal
**Step I + II**	66	15.2	step I: paracetamol, metamizole
			step II: tramadol*
**Step I + III**	152	35.1	step I: paracetamol, metamizole
			step III: morphine, oxycodone, fentanyl transdermal, pethidine, oxycodone-naloxone
**Step I + II + III** ^b^	84	19.4	step I: paracetamol, metamizole
			step II: tramadol*
			step III: morphine

^a^ NSAIDs = nonsteroidal anti-inflammatory drugs.

^b^ 58 cases with an overlap (≥ 24 hours) of opioids (step II and step III**).**

* Total: 147 cases including tramadol prescriptions.

### Coanalgesic and Concomitant Drugs

Overall, 43.4% (188) of all patients with chronic pain received drugs prescribed as coanalgesics. In addition, quite a number of these patients received drugs that were not primarily prescribed as analgesics but that have analgesic properties in some painful conditions (concomitant drugs) (Table 5).

**Table 5:** Classification of coanalgesics and concomitant drugs.

Drug class	Coanal-gesics			Con-comitant drugs			Number of cases	Reason for classification
**Alpha-2 adrenergic agonists**							4	**multipurpose**
Clonidine								hypertension
**Antidepressants**								**multipurpose**
Mirtazapine							38	66 cases with diagnosis of depression
Citalopram							24	84 cases without diagnosis of depression
Trazodone							17	
Escitalopram							15	
Venlafaxine							15	
Amitriptyline							13	
Trimipramine							12	
Sertraline							11	
Duloxetine							8	
Fluoxetine							5	
Olanzapine							3	
Clomipramine							2	
Paroxetine							2	
Mianserin							2	
St. John's wort preparations							2	
Opipramol							1	
**Antiemetic, prokinetic**								nausea, vomiting, prokinetic
Domperidone							367	
Metoclopramide							145	
**Antiepileptic drugs**								
Phenytoin							1	epilepsy
Carbamazepine							7	epilepsy; painful trigeminal neuropathy
Valproate							3	epilepsy
Pregabalin							32	peripheral neuropathy
Gabapentin							8	epilepsy; peripheral neuropathy
Topiramate							3	epilepsy; migraine
**Corticosteroids**								**multipurpose**
Prednisone							104	antiinflammatory, relief of different symptoms (e.g., pain, nausea, fatigue)
Dexamethasone							25	
Methylprednisolone							16	
Hydrocortisone							4	
**Baclofen**							3	spasticity and pain
**Benzodiazepines**								nervousness, sleep disturbance, anxiety disorders
Lorazepam							147	
Oxazepam							40	
Bromazepam							8	
**Butylscopolamine**							35	pain from bowel obstruction
**General Anesthetic**							2	
Ketamine								
**Local Anesthetic**								
Lidocaine							6	
Ropivacaine							3	
Capsaicin (topical)							2	
**Magnesium sulfate**								hypomagnesemia, constipation, spasms
Magnesium aspartate							84	
Magnesium citrate							2	
**Muscle relaxants**								exacerbation or acute musculoskeletal pain
Tizanidine							11	
Tolperisone							1	
**Laxatives**								prophylaxis and management of constipation
Liquid paraffin, combinations							382	(e.g., opioid induced)
Macrogol, combinations							119	
Lactulose							35	
Sodium picosulfate							31	
Senna glycosides							18	
Glycerol							10	
Bisacodyl							9	
Ispaghula (psyllium seeds)							7	
Lactitol							2	
Sodium phosphate							1	
**Neuroleptic drugs**								management of delirium, psychosis, nausea, emesis
Haloperidol							20	
Quetiapine							6	
Risperidon							4	
Chlorpromazine							3	
Olanzapine							3	
Paliperidone							1	
**Osteoclast inhibitors**								prevent skeletal-related events, may improve pain
*Bisphosphonate Derivative*								(neuropathic or malignant bone pain)
Alendronic acid							9	
Risedronic acid							1	
Calcitonin							9	

Antidepressants were assigned to both categories (coanalgesic and concomitant drugs). Potentially, antidepressants could be considered for the treatment of chronic pain and for the treatment of depressive disorders (66 cases). Alpha-2 adrenergic agonists, antidepressants, and corticosteroids have been categorized as multipurpose analgesics (multipurpose drugs have a various range of indications). Almost all patients received laxatives and antiemetic drugs. Most laxative agents were those that predominantly soften the stool (liquid paraffin). In addition to prokinetic agents/anti-emetics (domperidone and metoclopramide), other drugs ‒for example, corticosteroids ‒may enhance an antiemetic strategy. Pregabalin has been identified as a coanalgesic in the management of neuropathic pain (32 cases). All other antiepileptic drugs should be considered in patients with epilepsy or pain relief.

### Drug-Drug Interactions

With an average mean of 10.4 medications (SD = 4.9) prescribed per inpatient hospitalization with chronic pain, the amount of polypharmacotherapy (five or more drugs) was high, at 89% of all cases. We identified 3,186 potential DDIs (overlapping drug orders of drug pairs listed as interacting in the drug-drug interaction knowledge base), with 551 (17%) potential interactions between analgesics (without coanalgesics); 175 (5.5%) were with opioids. None of the interactions were classified as severity level 1 interactions (“contraindicated”). The DDIs knowledge base classified most potential interactions with opioids in our inpatients as severity level 3 (“monitoring or adaption required”); the only exception were the DDIs between buprenorphine and opioid agonists, as severity level 2 (“contraindicated as a precaution”) (Table 6). In a later version of this DDIs knowledge base, hospINDEX (2016–05), the interaction with buprenorphine is classified as severity level 3.

**Table 6:** Potential opioid related drug-drug interactions (DDIs) in multimorbid patients with chronic pain (n = 433), identified by using the galdat/hospINDEX® database. Multiple counts were allowed.

Medication class 1 (identified drugs) *	Medication class 2 (identified drugs) *	Potential DDI (n)	Severity level of inter-action^b^	Possible drug interaction
**Antidepressants: SSRIs, SNRIs**	**Opioids**	57	5	Provocation of a serotonin syndrome
(trazodone, escitalopram, venlafaxine, citalopram, duloxetine, sertraline, fluoxetine, and paroxetine)	(oxycodone, tramadol, and pethidine)			
**Anticoagulation drugs**	**Opioids**	32	3	Increased effect of anticoagulation drugs
(phenprocoumon)	(tramadol)			Risk of bleeding
**Azole antifungal agents**	**Opioids**	30	3	Increased analgesic effect
(fluconazole, itraconazole, and voriconazole)	(oxycodone, tramadol, and fentanyl)			(increased drug toxicity or prolonged adverse events)
**CYP 3A4 inducers**^a^	**Opioids**	25	3	Decreased analgesic effect
(rifampicin, carbamazepine, St. John's wort preparations [Hypericum perforatum], phenobarbital, and phenytoin)	(oxycodone, fentanyl, and tramadol)			
**Benzodiazepine**	**Opioids**	10	3	Increased risk of CNS depressant effects ^c^
(lorazepam, oxazepam, bromazepam)	(buprenorphine)			(hypotension, respiratory depression, or sedation)
**Opioid partial agonist drugs**	**Opioids**	8	2	Decreased analgesic effect
(buprenorphine)	(morphine, oxycodone, and tramadol)			Risk of withdrawal syndrome
**CYP 3A4 inhibitors**	**Opioids**	7	3	Increased effect of fentanyl derivatives
(verapamil, clarithromycin, amiodarone)	(fentanyl)			
**Antidepressants, tricylcic**	**Opioids**	6	3	Increased risk of seizures
(trimipramine, amitriptyline, opipramol)	(tramadol)			

DDIs with buprenorphin and opioid agonists: The level 2 of severity recommended by Galdat is controversially discussed in the literature [31].

^a^ CYP = cytochrome P450.

*based on their frequency.

^b^ Severity according to galdat/hospINDEX®: 1: ‘contraindicated’, 2:‘contraindicated as a precaution‘, 3: ‘monitoring or adaptation required’, 4: ‘monitoring or adaption in case of risk factors’, 5: ‘monitoring as a precaution’, 6: ‘no action required‘.

^c^ CNS = central nervous system.

Table 7 shows an overview of DDIs between analgesics, coanalgesics, concomitant drugs, and other drugs (e.g., antihypertensive drugs) prescribed to the study population of multimorbid inpatients with CP. All potential DDIs listed here were identified in the data set accordingly. This table is based on the recommendations in the drug-drug interactions knowledge base used in this study (galdat/hospINDEX®).

**Table 7:** Overview of the relevant drug interaction potential of analgesics (categorized into analgesics, coanalgesics, and concomitant drugs), identified by galdat/hospINDEX® database in the sample of chronic pain patients (n = 433). Antidepressants are included here as coanalgesics.

	Non-opioids		Opioids				Coanalgesics				
Overview of DDIs in multimorbid chronic pain patients	NSAIDs	Paracetamol	Tramadol	Oxycodone	Pethidine	Buprenorphine	Antidepressants, tricyclic	SSRIs, SNRIs	Local anesthetics (lidocaine)	Osteoclast inhibitors (bisphosphonates, calcitonin)	Anticholinergic drugs (e.g. scopolamine)
List of interacting drugs as recommended by knowledge base*											
Vitamin K antagonists (phenprocoumon)	3	4	3				5	5			
Antidepressants, tricylcic			3					3			3
Benzodiazepines						3					
Antiepileptic drug (phenytoin)											
Antiepileptic drug (valproate)											
Buprenorphine											
SSRIs, SNRIs	3		3	3	3		3				
Beta blocker (metoprolol)	4							5	5		
Magnesium salts										3	
Neuroleptic drugs: among others haloperidol							3	3			3
Serotinergic receptor agonists								5			
Diuretics (e.g loop diuretic)	4										
Antidiabetics: among others metformin	5										
Cardiac glycoside											
Dopamine receptor agonists: e.g. levodopa											
Corticosteroids, systemic	3										
Antiplatelet agents: among others acetylsalicylic acid	3										
Angiotensin-converting enzyme inhibitors	4										
Antifolates	3										
Osteoclast inhibitors: bisphosphonates, calcitonin											

			Concomitant drugs								
Corticosteroids, systemic	Alpha-2 adrenergic agonists	Neuroleptic drugs (e.g. haloperidol)	Antiepileptic drug (phenytoin)	Antiepileptic drug (carbamazepine)	Antiepileptic drug (valproate)	Anticonvulsant drug (topiramate)	Benzodia-zepines	Antiemetics, prokinetics (domperidone)	Antiemtics, prokinetics (metoclopramide)	Magnesium salts	Laxatives
											
5					5						
	3	3						3			
			5		3						
							5				
						3	3				
							3				
		3		3					3		
	3										
											
								3	3		
											
5											5
3											
5											4
		5									
			3	3							
											
											
											
										3	

SSRI, SNRIs: escitalopram, citalopram, sertraline, venlafaxine, duloxetine, fluoxetine, paroxetine.

Antidepressants, tricylcic: trimipramine, amitriptyline, clomipramine, mianserin.

Neuroleptic drugs: pipamperone, thiethylperazine, risperidon, chlorpromazine, quetiapine.

Benzodiazepines: lorazepam, oxazepam, bromazepam.

Pharmacokinetics: A range of drugs prescribed for our inpatients may be affected by induction or inhibition of mainly CYP3A4. These drugs are shown as examples in Table 6, with a focus on opioids.

* Severity according to galdat/hospINDEX® 1: ‘contraindicated’, 2:‘contraindicated as a precaution‘, 3: ‘monitoring or adaptation required’, 4: ‘monitoring or adaption in case of risk factors’, 5: ‘monitoring as a precaution’, 6: ‘no action required‘.

## Discussion

This study provides a general overview of the most common chronic pain diagnoses and combinations of chronic diseases (triplets) and the prescribed medications, respectively, in the multimorbid population under study. We discuss possible consequences of these data in regard to the impact of pain treatment on different multiple disease combinations found and vice versa. Furthermore, we discuss potential DDIs related to pain treatment with a focus on opioids and some selected coanalgesic or concomitant drugs.

In our study, 38% of all multimorbid inpatients had chronic pain. On average, these CP patients were 65.7 years old, had 6.6 chronic diagnoses, and were exposed to a high degree of polypharmacotherapy (10.4 drugs). The most common chronic pain diagnoses were musculoskeletal diseases (see Table 2). The most common triple disease combination was hypertension, chronic kidney disease, and chronic back pain (see Table 3). Opioids were prescribed in more than two thirds of CP patients for moderate to severe pain. Coanalgesic drugs were prescribed for 43% of these patients. The DDIs knowledge base classified most DDIs for these multimorbid patients with CP as severity level 3 (“monitoring or adaption required”).

According to the WHO analgesic ladder, non-opioids should be combined with opioids, but opioids should not necessarily be combined with other opioids. Some experts [32, 33] have recommended omitting the intermediate step of weak opioids completely and using low doses of strong opioids in combination with non-opioid analgesics instead. They argue in favor of this adaptation (a modified two-step approach) of the original WHO analgesic ladder mainly on the basis that it would establish more effective and simple pain therapy, in particular for patients with cancer. Omitting step II medication is likely to permit using only one single (strong) opioid, with modification in doses, and titration according to the patient’s individual pain level. In general, the use of opioids in combination with other opioids seems to be largely based on empirical evidence and depends on the physician’s clinical experience [34]. Furthermore, new formulations of analgesics have been developed in the past 10 years—for example, to prevent the most common side effect of opioid-induced constipation. Meanwhile, the co-administration of oral naloxone together with oxycodone (prolonged release) is a well-known regimen [35].

Tramadol in combination with other analgesics (in total 147 cases with tramadol prescriptions) was one of the most frequently prescribed analgesics. Tramadol has a specific dual mode of action relating to its affinities to the opioid receptors (modest affinity with μ-receptor, weak affinity to δ and κ) and/or the inhibition of the uptake of noradrenaline and serotonin [36]. Accordingly, tramadol may cause less constipation and respiratory depression than other strong opioids (e.g., morphine) and might therefore be an alternative choice in patients with multiple chronic conditions before more potent opioid analgesics are used. Tramadol can be especially useful in older patients who do not tolerate more potent opioids [37].

The common use of NSAIDs has been identified as critical, especially in terms of adverse drug events in elderly patients [38, 39]. NSAIDs pose three major risks: they may interfere with blood pressure control, aggravate renal impairment, or cause gastrointestinal bleeding. In this study, NSAIDs were found to be rarely used (only in 16%), probably to avoid such well-known interactions. In our study population chronic kidney disease was a common chronic condition, 29.1%. This may have been another reason for the low prescription rate of NSAIDs. At different stages of chronic kidney disease some opioids should be used with caution (risk of accumulation) possibly at reduced and less frequent doses [40]. However, buprenorphine seems to be a safe choice in patients with chronic kidney disease [40].

To date, the potential interaction risk of metamizole has not been sufficiently addressed in research [41]. The same is true for the impact of paracetamol on diagnosed hypertension [42]. But there is some evidence that paracetamol may lessen the effect of antihypertensive drugs [43]. By contrast, corticosteroids can have a particular impact in combination with diabetes, osteoporosis, and hypertension (e.g. increased blood pressure). Aggravations of diabetes mellitus, as a result of corticosteroid intake as an additional risk factor for osteoporosis are well-documented. Moreover, administering high doses of corticosteroids may trigger neuropsychiatric reactions [44], which may lead to an interaction with a coexisting mental health problem (e.g. psychosis caused by steroids).

In our study, 17% of total potential DDIs were identified as DDIs with analgesic prescriptions by galdat/hospINDEX®. In particular, this knowledge base put the main focus on drugs involved in the opioid-cytochrome P450 isoenzymes metabolism and multiple drug transporters (e.g., P-glycoprotein). These well-known DDIs are classified as inhibitors, inducers, or substrate accordingly (see Table 6; drugs as inducers/inhibitors). Some specific properties have to be noted here, in addition to the recommendation of this knowledge base, such as the fact that St. John’s Wort (SJW) preparations vary greatly and have different effects on CYP3A4 induction or in the drug transporter systems. SJW medicinal products with hyperforin-low *Hypericum perforatum* extracts are usually not considered to be of clinical relevance as inducers [45, 46]. A clinical pharmacokinetic study showed that treatment with rich-hyperforin SJW extracts decreases the plasma concentration of oxycodone [47].

For most clinical interactions with opioids identified in our study, galdat/hospINDEX® resulted in the following advice: “monitoring or adaptation required” (severity level 3). Still, for the use of the knowledge base a daily review of current medications is recommended (e.g., correct applications of drugs and dosing), as well as an individual risk assessment to enhance patient safety. However, the risk of interactions and adverse events may increase with every additional medical condition and every additional drug prescribed (polypharmacotherapy) [48, 49]. Table 5 (overview of coanalgesic and concomitant drugs), shows that concomitant drugs (laxatives, prokinetic/anti-emetics or neuroleptics) contribute to a high amount of polypharmacotherapy in this study. Potential DDIs are typically analyzed as drug-drug interaction pairs. For multimorbid patients with multiple drug and disease combinations, the analyses of two-way drug combinations are certainly not sufficient (see Table 7 for an overview of drug-drug interactions related to opioid prescriptions). For example, antidepressants (concomitant or even coanalgesic drugs) can imply an additive interaction risk, as these drugs, identified as having the most frequent DDIs with opioids in this study, have the potential for numerous drug interactions [50]. Mental disorders, such as depression, are common comorbid conditions in multimorbid patients. It is well known that the prevalence of depression doubles in patients with pain [51]. SSRIs (selective serotonin reuptake inhibitors) might interfere to various degrees with opioids but also with oral anticoagulation drugs (atrial fibrillation had a prevalence of 16.2% in this study) or even NSAIDs, resulting in a cumulative risk for adverse events [52].

Pain management is usually multimodal and in the first instance aims at reducing pain sufficiently and quickly. Beyond the desired effects of opioids, exogenous opioids can affect a number of the body’s own regulation systems, especially cardiac and vascular regulation [53].

Table 3 shows the most common chronic pain diagnoses and triplets of chronic conditions in our multimorbid inpatients. Musculoskeletal pain is not only a common chronic condition in elderly patients in primary care [54] but also an important coexisting condition in medical inpatients with multimorbidity, as shown by our study results. Overall, hypertension was the most common condition (57.7%). This was also reported, for multimorbid patients, with similar prevalence estimates, in a study of the most common disease combinations [55]. Other relationships may exist between hypertension and pain perception. Some studies suggest that chronic pain-related opioid changes (e.g., opioid receptor downregulation) such as hypoalgesia (i.e., decreased sensitivity to pain) are associated with elevated resting blood pressure [56]. These findings raise questions about potential modifying effects of opioids on pain sensitivity in patients with untreated hypertension or insufficiently lowered blood pressure. Similar considerations apply to other common disease combinations in multimorbid patients with CP, such as the treatment of diabetes mellitus (23.1% in this study) and its effect on chronic neuropathic pain. Endogenous opioids also have a role in the regulation of blood glucose and hyperinsulinemia [57], and it is conceivable that exogenous opioids, such as morphine, may affect this regulation.

### Implications for Research

Intensified research is needed on relevant interactions in clinical practice (e.g. pragmatic trials), which simultaneously includes multimorbid patients with polypharmacotherapy receiving analgesics, coanalgesics, and a substantial number of concomitant drugs. Research of this kind is scarce, and precise data on the interplay of interactions and polypharmacotherapy (on all sorts of modifying effects) are lacking. Furthermore, it is unclear how the interaction potential changes in the case of polypharmacotherapy and a reduction of drugs (deprescribing) [58]. A great need for research exists in this setting (e.g., increasing or decreasing drug dosages and omitting drugs or adding additional drugs, respectively). Reliable knowledge bases should be enlarged (interface options) with patient-specific static information (e.g., demographics, concurrent diseases) and dynamic information (e.g., lab results, vital signs), as well as relevant pharmacodynamic and pharmacokinetic aspects of co-medication and the time course of therapy (start, end, maintenance treatment) [59].

The implementation of a comprehensive tool for the assessment of potential DDIs and drug-disease interactions in multimorbidity, with a multimorbidity score to estimate the severity of potential interactions, is probably essential for well-structured, evidence-based clinical decision making. More research is needed to gain insights into harmful and beneficial effects of various interactions. In the context of this study, future research could shed light on the (positive and negative) effects of pain management on the body’s regulation systems—for example, in terms of possible drug-endogenous opioid activity interactions (e.g., exogenous opioids as possible ligands of the endogenous opioidergic regulation systems). Critically, the present findings indicate that multimorbidity is insufficiently well-managed by exclusively considering and treating the individual diseases of a patient with multiple chronic conditions. The potential interactions (DDIs, drug-multimorbidity interactions) have yet to be investigated.

Our study provides the basis for a deeper understanding of important interaction potential of patients with chronic pain. As a next step, frequent disease- and drug-combinations in multimorbidity should be investigated in pragmatic clinical trials.

### Limitations

Our study has several limitations. First, we examined a highly specific study population at a tertiary hospital. Our results are based on a retrospective medical chart review; with potential underreporting. Second, owing to the retrospective study design, chronic pain diagnoses had to be taken from medical health records without accompanying self-reports from patients—for example, by using the Visual Analog Scale for Pain [60, 61]. Third, we used the galdat/hospINDEX® knowledge base with country-specific information to identify potential DDIs. Fourth, we analyzed analgesic prescriptions and not medication intake. The interactions with opioids reported in this study will therefore have to be interpreted as potential medical conflicts and not as actual adverse events. A particular strength of our study was the use of detailed electronic health records for each patient, to review and complete the clinical administrative data.

## Conclusions

Our study explored retrospective data of multimorbid patients with chronic pain at a tertiary teaching hospital. To our knowledge this is the first study examining complex treatment situations in multimorbid patients with chronic pain. The following conclusions can be drawn:

Analgesics and analgesia-related drugs are often prescribed in combination with a large variety of other drugs, accompanied by a great potential of risks for multimorbid patients. Analgesic-related DDIs, in particular opioids, in multimorbid patients are often complex and difficult to assess by using DDI knowledge bases alone. Drug-multimorbidity interactions are not sufficiently investigated and understood. Currently, the scientific literature is scarce for chronic pain in combination with multiple coexisting medical conditions and medication regimens. Our work may provide useful information to enable further investigations in multimorbidity research within the scope of potential interactions and chronic pain.
